# Calretinin as a Marker for Premotor Neurons Involved in Upgaze in Human Brainstem

**DOI:** 10.3389/fnana.2015.00153

**Published:** 2015-12-14

**Authors:** Christopher Adamczyk, Michael Strupp, Klaus Jahn, Anja K. E. Horn

**Affiliations:** ^1^Department of Neurology, Klinikum Großhadern, Ludwig-Maximilians UniversityMunich, Germany; ^2^German Center for Vertigo and Balance Disorders, Klinikum Großhadern, Ludwig-Maximilians UniversityMunich, Germany; ^3^Department of Neurology, Schön Klinik, Bad AiblingGermany; ^4^Institute of Anatomy and Cell Biology, Dept. I, Ludwig-Maximilians UniversityMunich, Germany

**Keywords:** saccadic burst neurons, rostral interstitial nucleus of the medial longitudinal fascicle, interstitial nucleus of Cajal, y-group, oculomotor nucleus

## Abstract

Eye movements are generated by different premotor pathways. Damage to them can cause specific deficits of eye movements, such as saccades. For correlative clinico-anatomical post-mortem studies of cases with eye movement disorders it is essential to identify the functional cell groups of the oculomotor system in the human brain by marker proteins. Based on monkey studies, the premotor neurons of the saccadic system can be identified by the histochemical markers parvalbumin (PAV) and perineuronal nets in humans. These areas involve the interstitial nucleus of Cajal (INC) and the rostral interstitial nucleus of the medial longitudinal fascicle (RIMLF), which both contain premotor neurons for upgaze and downgaze. Recent monkey and human studies revealed a selective excitatory calretinin (CR)-positive input to the motoneurons mediating upgaze, but not to those for downgaze. Three premotor regions were identified as sources of CR input in monkey: y-group, INC and RIMLF. These findings suggest that the expression pattern of parvalbumin and CR may help to identify premotor neurons involved in up- or downgaze. In a post-mortem study of five human cases without neurological diseases we investigated the y-group, INC and RIMLF for the presence of parvalbumin and CR positive neurons including their co-expression. Adjacent thin paraffin sections were stained for the aggrecan (ACAN) component of perineuronal nets, parvalbumin or CR and glutamate decarboxylase. The comparative analysis of scanned thin sections of INC and RIMLF revealed medium-sized parvalbumin positive neurons with and without CR coexpression, which were intermingled. The parvalbumin/CR positive neurons in both nuclei are considered as excitatory premotor upgaze neurons. Accordingly, the parvalbumin-positive neurons lacking CR are considered as premotor downgaze neurons in RIMLF, but may in addition include inhibitory premotor upgaze neurons in the INC as indicated by co-expression of glutamate decarboxylase in a subpopulation. CR-positive neurons ensheathed by perineuronal nets in the human *y*-group are considered as the homolog premotor neurons described in monkey, projecting to superior rectus (SR) and inferior oblique (IO) motoneurons. In conclusion, combined immunostaining for parvalbumin, perineuronal nets and CR may well be suited for the specific identification and subsequent analysis of premotor upgaze pathways in clinical cases of isolated up- or downgaze deficits.

## Introduction

The ocular bulb is moved by six extraocular muscles that are controlled by motoneuron efferents from the oculomotor (nIII), trochlear (nIV) and abducens nucleus (nVI; for review, see Büttner-Ennever, 2006). Within the nIII the motoneurons of the medial rectus (MR), superior rectus (SR), inferior rectus (IR) and inferior oblique (IO) are arranged in a topographic manner. The IR subgroup is located dorsomedially in the rostral nIII, while the central part of nIII contains the motoneurons of SR and IO, both mediating upgaze. In primates, the MR motoneurons are represented in two subgroups, the B-group in the dorsolateral part of the caudal nIII and the A-group in the ventral nIII (Büttner-Ennever, 2006; Büttner-Ennever and Horn, 2014; Che Ngwa et al., 2014).

The SR and the IO motoneurons mediate upward eye movements (Leigh and Zee, 2015). Tract-tracing studies in monkey have shown that the motoneurons of the ipsilateral IO and contralateral SR lie intermingled within the central part of the caudal half of the nIII (Büttner-Ennever, 2006; Zeeh et al., 2013). Recent studies in monkeys demonstrated that the motoneurons of SR and IO receive a selective input from nerve endings containing the calcium-binding protein calretinin (CR; Zeeh et al., 2013). This CR-input was confined to motoneurons involved in upgaze and included the motoneurons of the levator palpebrae muscle (LP), which show a similar activity pattern to that of the SR except during blinks (Fuchs et al., 1992; Evinger and Manning, 1993). Simultaneous immunostaining for CR and GABAergic markers that had lacked any colocalization suggested that the premotor CR-positive input to upgaze motoneurons is excitatory (Zeeh et al., 2013). Combined tract-tracing and immunohistochemical staining for CR in monkeys revealed three major brainstem sources of CR-positive inputs to upgaze motoneurons: the rostral interstitial nucleus of the medial longitudinal fascicle (RIMLF), the interstitial nucleus of Cajal (INC) and the vestibular y-group (Ahlfeld et al., 2011). The RIMLF contains premotor burst neurons encoding vertical, either upward or downward, and torsional saccades (Büttner et al., 1977). Recordings and anatomical studies in monkeys revealed that upward and downward premotor neurons are intermingled within the RIMLF (Moschovakis et al., 1991a,b; Horn and Büttner-Ennever, 1998). The INC contains several functional cell groups involved in vertical eye and head movements. Excitatory and inhibitory premotor neurons in the INC are thought to integrate the eye velocity signals received from the RIMLF into eye position signals of the motoneurons reflected in their burst-tonic firing pattern (Leigh and Zee, 2015). The vestibular y-group receives disynaptic inputs from vertical canal afferents (Blazquez et al., 2000) and projects directly to motoneurons in nIII and nIV (Carpenter and Cowie, 1985; Wasicky et al., 2004). It may be involved in the generation of vertical smooth-pursuit eye movements (Chubb and Fuchs, 1982) and modulation of the vertical vestibulo-ocular reflex (VOR). All three brainstem regions have been described in the human brainstem by their cytoarchitectural and histochemical features (Horn and Adamczyk, 2011; Büttner-Ennever and Horn, 2014). In the human RIMLF premotor saccadic burst neurons can be identified by their association with a specialized extracellular matrix known as perineuronal nets and the expression of the calcium-binding protein parvalbumin (PAV; Horn and Büttner-Ennever, 1998; Horn et al., 2003a).

In correspondence to the findings in monkeys, a recent post-mortem study in human tissue revealed that the central caudal nucleus containing LP motoneurons and the central portion of nIII receive a selective input from CR-positive nerve fibers. In accordance with the monkey data this central nIII group is now considered to be the location of SR and IO motoneurons in humans (Che Ngwa et al., 2014). Thus immunostaining for CR may provide a histochemical tool to identify excitatory premotor neurons for upgaze in the human brainstem. To address this issue we performed a post-mortem study in humans to investigate whether the RIMLF, INC and y-group contain CR-positive neurons, which meet the criteria of functional cell groups of the vertical eye movement system.

## Materials and Methods

### Antisera

#### Parvalbumin (PAV)

The mouse monoclonal antibody against PAV (clone 235; Swant, Switzerland) was raised against PAV that had been purified from carp muscles (Celio et al., 1988). This PAV antibody specifically stains the ^45^Ca-binding spot of parvalbumin (MW 12,000 and IEF 4.9) in a two-dimensional immunoblot.

#### Calretinin (CR)

For the detection of CR in neuronal profiles a rabbit polyclonal CR antibody (7699/3H, LOT 18299, Swant, Bellinzona, Switzerland) was used. CR is a calcium-binding protein of the EF-hand family, related to calbindin D-28k and calmodulin, with a widespread distribution within the brain in different species (Andressen et al., 1993). The CR antiserum was produced in rabbits by immunization with recombinant human CR containing a 6-his tag at the N-terminal.

#### Aggrecan (ACAN)

A monoclonal mouse anti-human ACAN core protein (ACAN; clone HAG7D4; Acris; Herford, Germany; SM1353P) was used to detect perineuronal nets. The immunogen was purified human articular cartilage aggrecan.

#### Glutamic Acid Decarboxylase (GAD)

GABAergic terminals were detected with a monoclonal antibody against the GABA-synthetizing enzyme GAD (GAD_65/67_ GC3108, batch number Z05507, clone 1111, Biotrend, Cologne, Germany). The antibody GC 3108 recognizes a linear epitope at the C-terminus of rat GAD, common to both isoforms. The hybridoma secreting the antibody to GAD_65/67_ was generated by fusion of splenocytes from a mouse immunized with fragments of recombinant human GAD_65_ fused to glutathione-S-transferase (Ziegler et al., 1996).

### Cases

The brainstems from five *post mortem* human cases (cases 1–5, Table 1) were obtained 24–72 h after death through the Reference Center for Neurodegenerative Disorders of the Ludwig-Maximilians University with written consent from next of kin, who confirmed the wishes at time of death. All procedures were approved by the Local Research Ethics Committees. The study is in accordance with the ethical standards laid down in the 1964 Declaration of Helsinki. The age of the donors ranged from 62–75 years, and there was no history of neurological disease. Case 1 was a 71-year-old male who died of heart failure after pneumonia whose neuropathological examination demonstrated considerable atherosclerosis with mild stenosis of brainstem vessels and mild frontal and temporal lobe atrophy. Case 2 was a 75-year-old female who died of heart failure after pneumonia whose neuropathological examination demonstrated a small infarct in the occipital white matter, arteriosclerosis, and stage I Alzheimer changes. Case 3 was a 62-year-old male who had died of pancreatic cancer without brain metastases or hepatic encephalopathy. His neuropathological examination revealed small old hemorrhages in the adenohypophysis, arteriosclerosis, and Braak and Braak stage I Alzheimer changes. Case 4 was a 67-year-old male with rectal cancer who died of heart failure whose neuropathological examination showed old infarcts in the right occipital and frontal lobe. Case 5 was a 75-year-old male with arteriosclerosis, who died of cardiac infarction.

**Table 1:** **List of human post-mortem cases used in the study**.

Case	Age, gender	Cause of death	Post-mortem delay (hours)	Fixation duration (days)
1	71, male	Multiple organ failure	24	7
2	75, female	Septic shock	24	2
3	62, male	Pancreatic cancer	24	6
4	67, male	Left heart failure	24	10
5	75, male	Cardiac infarction	72	10

*List of specimens, with gender, age, cause of death, post-mortem delay of fixation process and duration of fixation*.

### Human Tissue

The tissue was immersed in 10% formalin for 7–10 days. Blocks of the medulla and midbrain were embedded in paraffin, and serial sections of 5 μm and 10 μm thickness were cut from each case. Sections of 10 μm thickness were used for cresyl violet staining, and neighboring sections of 5 μm thickness were used for immunostaining for CR, PAV and ACAN to detect perineuronal nets. Additional selected sections were studied for the presence of GABAergic neurons by immunostaining for glutamate decarboxylase (GAD). Sections were deparaffinated in three changes of xylene, rehydrated in decreasing concentrations of alcohol (100%, 96%, 90%, and 70%) and rinsed in distilled water. Prior to immunostaining an antigen retrieval procedure was carried out by incubating the sections in 0.01 M sodium citrate buffer (pH 8.5) in a waterbath at 80°C for 15 min, and then for another 15 min at room temperature, before rinsing them (Jiao et al., 1999). Alternatively, sections were boiled for 3 × 10 min in a microwave in 0.01 M citrate buffer (pH 6) before the slides were transferred to 0.1 M phosphate-buffered saline (PBS; pH 7.3). Then, sections were treated with 3% hydrogene peroxide and 10% methanol for 15 min to block endogenous peroxidase activity. After buffer washes with 0.1 M Tris-buffered saline (TBS; pH7.4) sections were incubated in either 2% normal goat serum (for CR) or 2% normal horse serum (for PAV and ACAN) in 0.3% Triton-X100 in 0.1 M TBS for 1 h at room temperature to block non-specific binding sites. Sections were subsequently treated either with monoclonal mouse antibodies against the calcium-binding protein PAV (Swant, Marly, Switzerland; 1:2500) or against human ACAN core protein (ACAN; clone HAG7D4; Acris; Herford, Germany; 1:75) or with polyclonal rabbit antibodies against CR (Swant, Marly, Switzerland; 1:2500) for 2 days at 4°C. After washing in 0.1 M TBS, the sections were incubated either in biotinylated horse anti-mouse IgG (1:200;Vector Laboratories) or biotinylated goat anti-rabbit IgG (1:200; Vector Laboratories) at room temperature for 1 h, followed by three washes in 0.1 M TBS. Then, sections were incubated in extravidin-peroxidase (EAP; 1:1000; Sigma) for 1 h at room temperature. After two rinses in 0.1 M TBS, and one rinse in 0.05 M Tris-buffer (TB), pH 8, the EAP complex indicating the antigenic sites was visualized by a reaction in 0.025% diaminobenzidine, 0.4% ammonium nickel sulfate and 0.015% hydrogen peroxide in 0.05 M TB, pH 8, for 10 min. For the first runs of immunostaining nickel sulfate was omitted revealing a brown reaction product. Furthermore the reaction time in DAB with and without nickel sulfate was varied between 3 and 10 min to achieve optimal staining results. After washing, the sections were air-dried, dehydrated in alcohol, and coverslipped with DePex mounting medium (Sigma, St. Louis, MO, USA).

### Analysis

All slides were examined with a light microscope Leica DMRB (Bensheim, Germany). Neighboring sections containing RIMLF, INC or vestibular nuclei with y-group were imaged using a slide scanner (Mirax MIDI, Zeiss) equipped with a plan Apo-chromate objective (×20). The digitized images were viewed on a computer with the free software Pannoramic viewer (3D Histech; 1.152.3) at the same zooming magnification. The corresponding detailed views of equally arranged and magnified images were analyzed on the computer screen. Identified CR-positive neurons within the regions of interest were analyzed for the presence of PAV or GAD in the magnified image of the neighboring section using blood vessels as landmarks. Single and double labeled neurons were plotted on the outlines of the respective nuclei of interest with drawing software (Coreldraw 11.0; COREL). The same software was used to label the figures. High power photographs of examples were taken with a digital camera (Pixera Pro 600ES, Klughammer, MarktIndersdorf, Germany) mounted on the microscope and processed with Photoshop 7.0 software (Adobe Systems, Mountain View, CA, USA). The sharpness, contrast, and brightness were adjusted to reflect the appearance of the labeling seen through the microscope.

## Results

In all cases the immunostaining for the calcium-binding proteins CR and PV revealed a consistent staining pattern when using either one of the antigen retrieval procedures. Although there were slight differences in the staining intensity between different runs of the immunostaining procedure, especially when using the nickel intensified DAB reaction, the main pattern of immunopositive and immunonegative cells was similar. The most variable staining intensity was found for the detection of ACAN. Only weak ACAN-staining was found for case 2, whose brainstem was fixed only for 2 days, whereas all other cases, including case 5 with a post-mortem delay of 72 h, revealed robust immunostaining for all antibodies used in the study.

### Oculomotor Nucleus

**Figure 1:**
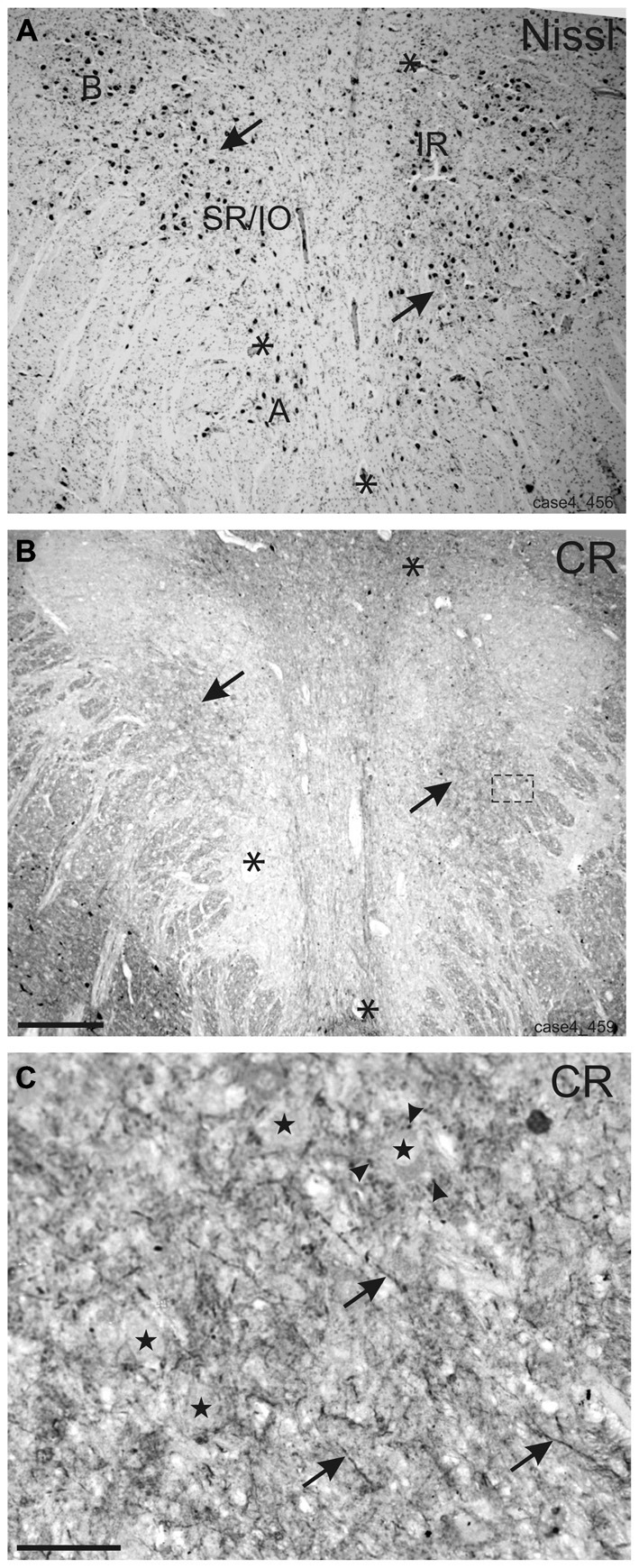
**Overviews of neighboring transverse sections through the human oculomotor nucleus with Nissl-staining (A) and calretinin (CR) immunostaining (B)**. For clarity corresponding blood vessels are indicated by asterisks **(A,B)**. In Nissl-staining the cytoarchitecture of nIII is demonstrated with its different motoneuronal subgroups **(A)**. Only the central subgroup of nIII receives a strong input from CR-positive fibers and terminals (**B**, arrows). The detailed view of the inset in **(B)** shows CR-positive fibers (**C**, arrows) and putative terminals (**C**, arrowheads) targeting putative superior rectus and inferior oblique motoneurons (**C**, stars). **A**: A-group of medial rectus motoneurons; **B**: B-group of medial rectus motoneurons; SR: superior rectus muscle; IO: inferior oblique muscle. Scale bar in **(B)** = 500 μm (applies to **A–B**); in **(C)** = 50 μm.

In Nissl staining, different subgroups can be identified in the human oculomotor nucleus (Figure 1A). The immunostained neighboring section demonstrates that strong CR expression is confined to the central group of nIII considered to be the motoneuronal subgroup of SR and IO muscle (SR/IO; Figure 1B, arrows). The detailed view within the SR/IO subgroup reveals numerous CR-positive fibers (Figure 1B rectangle, Figure 1C, arrows) and puncta (arrowheads) around motoneurons (Figure 1C, stars).

### Rostral Interstitial Nucleus of the Medial Longitudinal Fascicle

**Figure 2:**
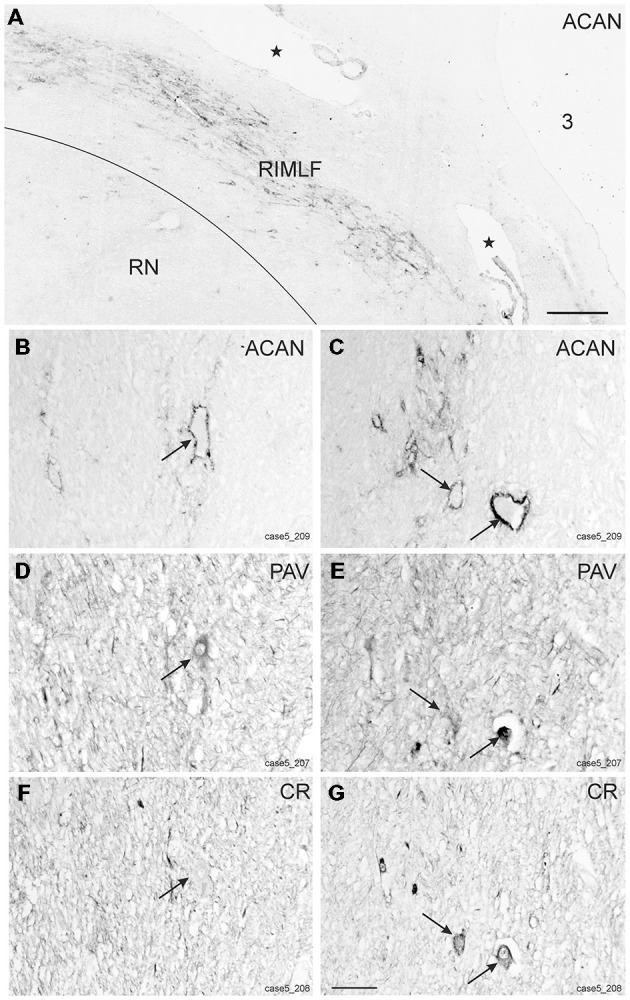
**Detailed views from transverse sections through the rostral midbrain at the level of the rostral interstitial nucleus of the medial longitudinal fascicle (RIMLF)**. The RIMLF is outlined by the presence of perineuronal nets here stained for aggrecan (ACAN) as a wing-shaped nucleus curving around the dorsomedial border of the red nucleus (RN) and bordered medially by the thalamo-subthalamic paramedian artery (**A**, stars). Magnifications of corresponding detailed views demonstrate examples of putative down-burst neurons, which are ensheathed by ACAN-based perineuronal nets (**B**, arrow), express parvalbumin (PAV)—immunoreactivity (**D**, arrow), but lack calretinin (CR; **F**, arrow). **(C–G)** Example of putative up-burst ACAN- and PAV-positive neurons (**C,E**, arrows) that express CR-immunostaining in addition (**G**, arrows). 3: third ventricle. Scale bar in **(A)** = 500 μm; in **(G)** = 100 μm (applies to **B–G**).

**Figure 3:**
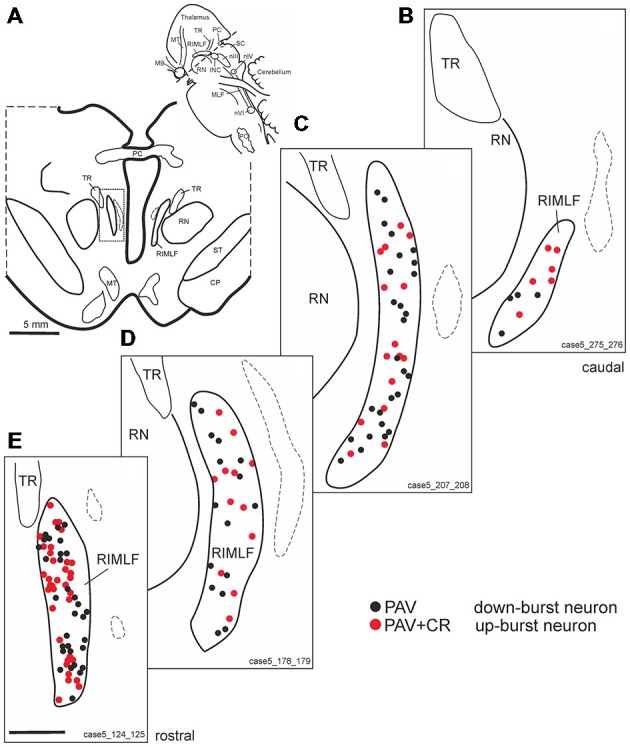
**(A)** Schematic transverse section through the rostral interstitial nucleus of the medial longitudinal fascicle (RIMLF) indicating the plane (see cutting angle in sagittal view in inset) and area of the plots shown in **B–E** (rectangle). Schematic drawings from the RIMLF at four different planes from caudal to rostral **(B–E)** demonstrating putative up-burst neurons identified by simultaneous expression of parvalbumin (PAV) and calretinin (CR; red dots) and down-burst neurons expressing PAV only (black dots). The thalamo-subthalamic artery serves as a useful landmark (**B–E**, dotted lines). Note that both populations are intermingled. CP, cerebral peduncle; INC, interstitial nucleus of Cajal; PO, principal inferior olive; MLF, medial longitudinal fascicle; nIII, oculomotor nucleus; nIV, trochlear nucleus; nVI, abducens nucleus; PC, posterior commissure; MB, mammillary body; MT, mammillothalamic tract; RN, red nucleus; SC, superior colliculus; ST, subthalamus; TR, tractus retroflexus. Scale bar in **(E)** = 1 mm (applies to **B–E**).

Within the rostral mesencephalon the wing-shaped RIMLF was identified by its strong rather selective labeling for perineuronal nets, here identified by detection of the extracellular matrix protein ACAN (Figures 2A–C). The RIMLF lies ventromedial to the third ventricle, embedded in the rubral capsule surrounding the rostral parvocellular red nucleus (RN), medially bordered by the thalamo-subthalamic paramedian artery (Figure 2A, stars, Figure 3A; Horn and Büttner-Ennever, 1998). Within the RIMLF we found medium-sized CR-positive neurons (Figure 2G) that were distributed evenly across the whole region of the RIMLF. There was no obvious distribution pattern for the CR neurons (Figures 3B–E).

Based on the fact that saccadic burst neurons express the calcium-binding protein PAV and are associated with strong perineuronal nets (Horn and Büttner-Ennever, 1998; Horn et al., 2003a) a careful investigation of neighboring sections stained for ACAN, PAV or CR was conducted for co-expression of PAV and CR. The analysis revealed that approximately 45% of PAV- and ACAN-positive neurons express CR (Figures 2C,E,G), whereas 54% showed only PAV-immunoreactivity (Figures 2B,D,F). Both populations of CR/PAV and PAV-positive neurons were intermingled throughout the whole extent of the RIMLF (Figures 3B–E).

### Interstitial Nucleus of Cajal

**Figure 4:**
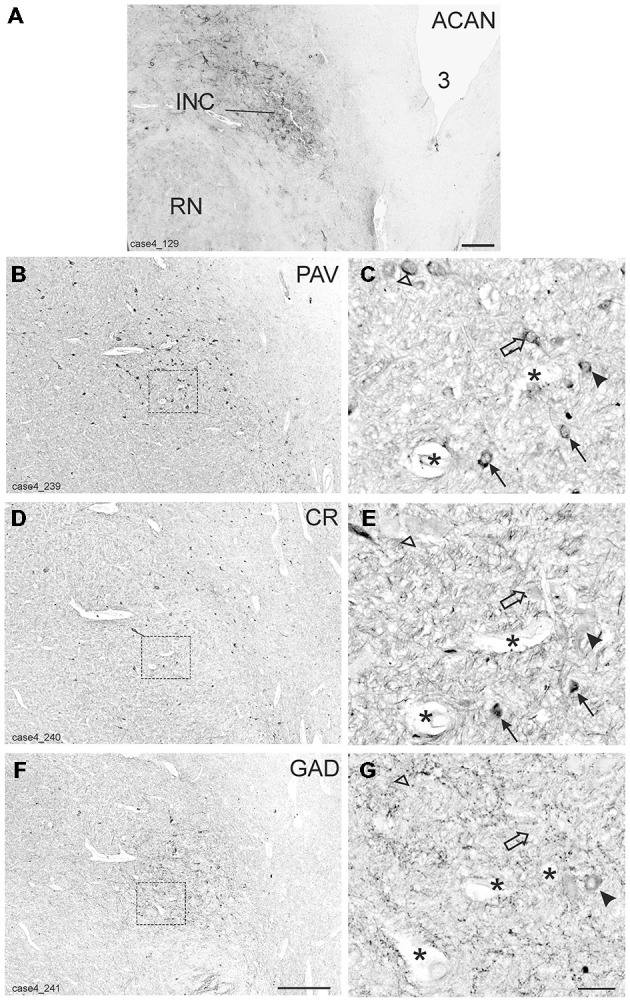
**Detailed views from transverse sections through the rostral midbrain at the level of the interstitial nucleus of Cajal (INC)**. The INC is highlighted by the presence of aggrecan (ACAN) -based perineuronal nets as a compact oval nucleus **(A).** Neighboring sections were stained for parvalbumin (PAV), calretinin (CR) and glutamate decarboxylase (GAD; **B,D,F)**. Rectangles indicate the areas for detailed views in **(C,E,G)**, which demonstrate examples of putative up-burst neurons, which contain parvalbumin (PAV) and calretinin (CR; **C,E**, arrows) and putative down-burst neurons, which express PAV, but lack CR (**C,E**, open arrows). In one example the additional lack of GAD could be demonstrated (**C,E,G** open arrow head) . An example of a putative inhibitory down-burst neuron, which expresses immunoreactivity for GAD and PAV, but lacks CR is indicated by an arrow head **(C,E,G)**. For clarity corresponding blood vessels are marked by asterisks. 3: third ventricle. Scale bar in **(A)** = 500 μm; in **(F)** = 500 μm (applies to **B,D,F**); in **(G)** = 50 μm (applies to **C,E,G**).

**Figure 5:**
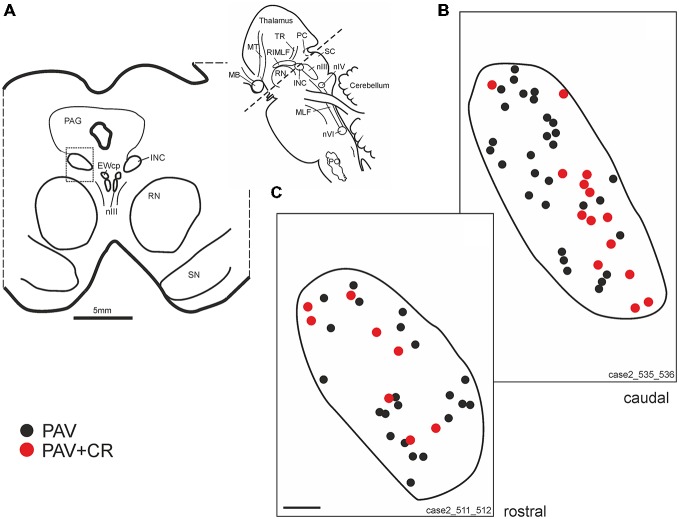
**(A)** Schematic transverse section through the interstitial nucleus of Cajal (INC) indicating the plane (inset) and area (rectangle) of the plots shown in **(B,C)**. Drawings of INC at two different planes from caudal and rostral INC **(B,C)** demonstrating putative upgaze neurons identified by simultaneous expression of parvalbumin (PAV) and calretinin (CR, red dots). Neurons expressing PAV only (**B,C**, black dots) may include excitatory and inhibitory downgaze neurons (compare Figures 4C,E,G, solid arrow head). Note that both populations are intermingled. EWcp: central projecting Edinger-Westphal nucleus; PO: principal inferior olive; MB: mammillary body; MLF: medial longitudinal fascicle; MT: mammillothalamic tract; nIII: oculomotor nucleus; nIV: trochlear nucleus; nVI: abducens nucleus; PAG: periaqueductal gray; PC: posterior commissure; RN: red nucleus; SC: superior colliculus; SN: substantia nigra.; ST: subthalamus; TR: tractus retroflexus. Scale bar in **(C)** = 200 μm (applies to **B,C**).

The INC lies among the fibers of the medial longitudinal fasciculus (MLF) in the rostral mesencephalic tegmentum and is easily identified in Nissl-stained sections as a compact nucleus composed of small to medium-sized neurons (Horn and Adamczyk, 2011; Büttner-Ennever and Horn, 2014; Figure 5A). It was highlighted by its strong ACAN- and PAV-immunolabeling (Horn and Adamczyk, 2011; Figures 4A,B).

As for RIMLF, the systematic analysis of adjacent 5 μm thick sections stained for either one of the calcium-binding proteins revealed a population of neurons expressing both CR and PAV (Figures 4C,E, arrows; Figures 5B,C, red dots), and neurons containing PAV only (Figures 4C,E, open arrows; Figures 5B,C, black dots). PAV-expression was found in small, medium-sized and large neurons within INC, whereas CR-immunoreactivity was present in small and medium-sized neurons. A coexpression of CR and PAV was mainly found in medium-sized neurons (Figures 4C,E). Both neuron groups were intermingled within the INC (Figures 5B,C). The additional analysis of neighboring sections in INC stained for GAD revealed that most CR-positive neurons lacked GAD, but it was found in a subpopulation of neurons containing PAV only (Figures 4C–G, arrow head).

### Vestibular y-Group

**Figure 6:**
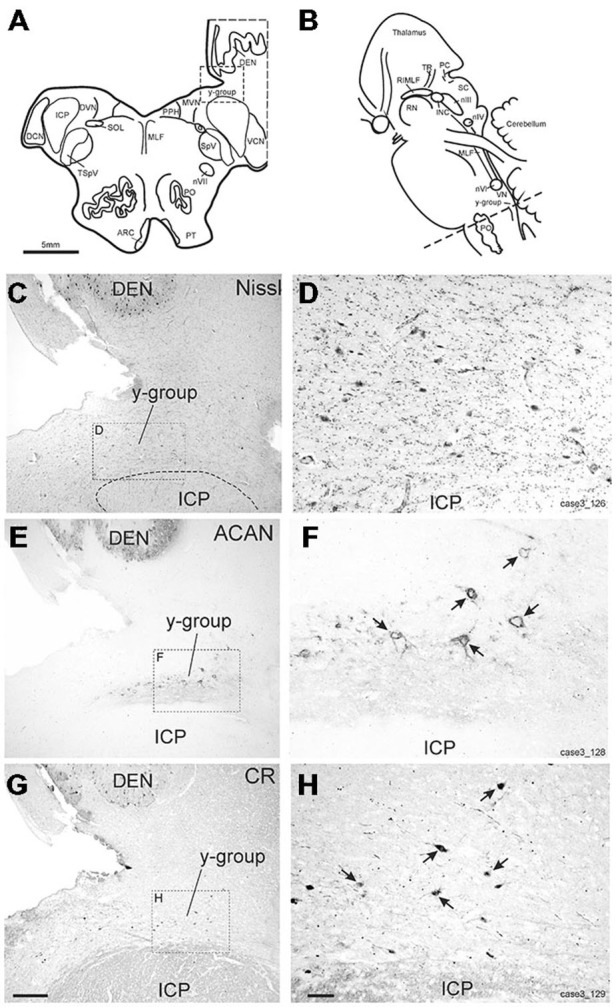
**(A,B)** Schematic drawings of a human brain stem, where the dotted line indicates the level of the neighboring transverse sections in **(C–H)**, **(C,E,G)** show overviews of transverse sections in cresyl violet staining to demonstrate the cytoarchitecture **(C)** immunostaining for aggrecan (ACAN) to identify the y-group (Y) **(E)** and for calretinin (CR) **(G)**. Rectangles indicate the detailed views shown in **(D,F,H)**. Note that most CR-positive neurons are associated with ACAN-based perineuronal nets as indicated by arrows pointing to corresponding neurons **(F,H)**. ARC, arcuate nucleus; DCN, dorsal cochlear nucleus; DEN, dentate nucleus; DVN, descending vestibular nucleus; ICP, inferior cerebellar peduncle; INC, interstitial nucleus of Cajal; PO, principal inferior olive; MLF, medial longitudinal fascicle; MVN, medial vestibular nucleus; nIII, oculomotor nucleus; nIV, trochlear nucleus; nVII, facial nucleus; PC, posterior commissure; PPH, prepositus nucleus; PT, pyramidal tract; RIMLF, rostral interstitial nucleus of the MLF; RN, red nucleus; SC, superior colliculus; SOL, solitary nucleus; SpV, spinal trigeminal nucleus; TR, tractus retroflexus; VCN, ventral cochlear nucleus; VN, vestibular nuclei. Scale bar in **(G)** = 500 μm (applies to **C,E,G**); in **(H)** = 100 μm (applies to **D,F,H**).

The y-group lies at the cerebellomedullary junction, bordered by the dorsal acoustic striae, the inferior cerebellar peduncle (ICP) and the cerebellar white matter containing the floccular peduncle (Büttner-Ennever and Horn, 2014; Figures 6A–D). Within the cerebellomedullary junction the y-group was identified by its staining pattern for perineuronal nets (ACAN; Figures 6E,F). Within the dorsal part of the y-group we found several medium-sized neurons with strong positive labeling for CR (Figures 6G,H). These CR positive neurons were surrounded by strong ACAN positive perineuronal nets (Figures 6F,G, arrows).

## Discussion

In accordance with the findings in the monkey (Ahlfeld et al., 2011), in humans the three premotor brainstem areas of the oculomotor system, RIMLF, INC and y-group contained medium-sized neurons that express immunoreactivity for the calcium-binding protein CR. We think that these CR-expressing neurons for the most part represent excitatory premotor neurons of the upgaze system for the following reasons. First, in the monkey, retrogradely labeled motoneurons of the SR, IO (and LP) were shown to receive a selective input from CR-immunoreactive terminals, not present around motoneurons involved in horizontal or downgaze (Zeeh et al., 2013). Second, inhibitory inputs to twitch motoneurons within nIII involved in vertical gaze are mediated only via GABAergic synapses, and not glycinergic inputs. The latter are confined to the motoneurons of horizontal eye movers (Spencer et al., 1989, 2003; Horn et al., 2003b; Zeeh et al., 2015). Therefore, a study of a possibly inhibitory nature of CR-positive terminals attaching upgaze motoneurons was restricted to GABA. Accordingly the lacking coexpression of CR- and GAD-immunoreactivity in nerve endings around upgaze motoneurons was taken as evidence that the CR input to these motoneurons is excitatory (Zeeh et al., 2013). Like in the monkey, in human nIII a selective CR input is confined to the central motoneuronal group, which is therefore considered to be the location of SR and IO motoneurons (Che Ngwa et al., 2014). Likewise the CR-positive afferents targeting SR and IO motoneurons are assumed to originate from CR-positive excitatory premotor cell bodies involved in upgaze. Given the similarities in histochemical properties of functional cell groups of the oculomotor system in the brainstem of the monkey and man (Horn, 2006; Horn and Adamczyk, 2011) we focused our analysis on CR-positive neurons in those premotor areas that actually have been proven to project to upgaze motoneurons in the monkey (Ahlfeld et al., 2011).

### Rostral Interstitial Nucleus of the Medial Longitudinal Fascicle

Recordings, stimulations and anatomical studies in the monkey showed that the RIMLF contains premotor burst neurons that encode vertical and torsional saccades (Büttner et al., 1977; Hepp et al., 1989; Horn, 2006). These neurons exhibit a high-frequency burst of up to 1000 spikes/seconds, shortly before and during vertical saccades (Büttner et al., 1977; Henn et al., 1984), which is in line with their association with prominent perineuronal nets (Horn et al., 2003a). Combined tract-tracing and immunostaining in the monkey revealed that all putative premotor saccadic burst neurons in the RIMLF express the calcium-binding protein PAV (Horn and Büttner-Ennever, 1998) and a large population in addition CR (Horn et al., 2003a; Ahlfeld et al., 2011). With the present knowledge of a selective CR input to only upgaze motoneurons, the CR-positive neurons can now be considered as the excitatory premotor up-burst neurons, and those expressing PAV only represent excitatory down-burst neurons (Ahlfeld et al., 2011). The fact that similar populations of PAV-positive neurons, some of them co-expressing CR, and all ensheathed by perineuronal nets, were identified in the human RIMLF also argues for the concept that CR serves as a histological marker for excitatory saccadic upward burst neurons in this nucleus. Likewise, neurons ensheathed by perineuronal nets but expressing PAV only represent putative excitatory saccadic down-burst neurons. As in previous physiological and anatomical studies in the monkey a specific spatial distribution of presumed excitatory up-burst and down-burst neurons was not detected in the human RIMLF (Büttner et al., 1977; Horn and Büttner-Ennever, 1998; Ahlfeld et al., 2011), whose structural lesions could explain the cause of an isolated up- or downgaze palsy in patients (Büttner-Ennever et al., 1982; Pierrot-Deseilligny et al., 1982; Leigh and Zee, 2015).

### Interstitial Nucleus of Cajal

The INC is known to play a major role in the neural integration of vertical and torsional eye movements (Fukushima et al., 1992; Leigh and Zee, 2015). The neural substrates for the velocity-to-position integration in the INC are the burst-tonic and the tonic neurons, which receive projections from burst neurons in the RIMLF (Moschovakis et al., 1991a,b) and project directly to motoneurons for vertical eye movements (Fukushima et al., 1992; Helmchen et al., 1996; Dalezios et al., 1998; Horn and Büttner-Ennever, 1998; Chen and May, 2007). Unlike the RIMLF, the INC contains more functional groups of saccade-related neurons, which may also be contained in our populations of PAV-immunoreactive neurons ensheathed with perineuronal nets based on their high firing rates. These include saccadic burst neurons (one third of all eye-movement related neurons), which show identical properties to those in the RIMLF (Helmchen et al., 1996). This population may involve premotor inhibitory upward neurons (Sugiuchi et al., 2013) and non-premotor downward burst neurons which project back to the ipsilateral RIMLF, believed to establish a feedback loop delivering a recurrent eye displacement signal to the RIMLF (Moschovakis et al., 1991a,b; Helmchen et al., 1996). Since CR is not present in GABAergic terminals in monkey nIII (Zeeh et al., 2013), premotor GABAergic neurons believed to inhibit the motoneurons of antagonistic muscles during vertical saccades (Horn et al., 2003b; Sugiuchi et al., 2013) are most likely not included in the CR-positive population in the human INC of the present study. Taken together, although the calcium-binding proteins CR and PAV may not represent exclusive markers for premotor up and down neurons in humans, the following assumptions can be made: (1) The CR/PAV-positive population is believed to contain excitatory premotor burst, burst-tonic neurons involved in upgaze. (2) The population expressing PAV only, but not CR, may include excitatory premotor neurons for downgaze. (3) Of the latter population those neurons expressing PAV only and GAD, may represent premotor inhibitory upward neurons, which project to the motoneurons of antagonistic eye muscles, e.g., IR and superior oblique. Such a GABAergic projection from the INC to the contralateral IR and superior oblique motoneurons has been demonstrated in the monkey (Horn et al., 2003b). The lack of a spatial separation of putative premotor upgaze and downgaze neurons within INC in the present anatomical work is in line with the observations from recording studies in the monkey (Fukushima et al., 1990; Helmchen et al., 1996).

### Vestibular y-Group

In accordance with the monkey a prominent population of CR-positive neurons was found in the vestibular y-group in humans not described previously. As in the monkey the y-group sits on the caudal slope of the ICP separated by the fibers of the acoustic striae (Büttner-Ennever and Gerrits, 2004; Büttner-Ennever and Horn, 2014). We believe that the CR-positive neurons in the present study correspond to those found in the monkey that project to the oculomotor nucleus (Ahlfeld et al., 2011) for the following reasons: the tracer-labeled CR-positive neurons in the y-group in the monkey showed a similar morphology to those seen in the human y-group in the present study (Büttner-Ennever and Gerrits, 2004). Likewise, the pattern of perineuronal net labeling corresponds to that found in monkey (Horn: own observations), and differs clearly from that described in the adjacent dorsal cochlear nucleus (Wagoner and Kulesza, 2009). The y-group receives a polysynaptic input from vertical canal afferents via interneurons in the superior and medial vestibular nuclei (Blazquez et al., 2000) and is targeted by direct inhibitory projections from ipsilateral floccular purkinje cells (Sato and Kawasaki, 1987), most of them coding for downward eye movements (Partsalis et al., 1995; Krauzlis and Lisberger, 1996). Accordingly, electrical stimulation of the y-group results in EPSPs in the IO and SR subgroups and slow upward eye movements (Chubb and Fuchs, 1982; Sato and Kawasaki, 1987). Likewise, an ipsilateral projection from the y-group to superior oblique and IR motoneurons is believed to be inhibitory (Sato and Kawasaki, 1987; Wasicky et al., 2004) and may be represented by GABAergic neurons in the y-group, which lack CR (Büttner-Ennever and Gerrits, 2004). Taken together the CR-positive neurons in the y-group may be involved in the generation of upward smooth pursuit eye movements and the modulation of the VOR (Chubb and Fuchs, 1981, 1982; Partsalis et al., 1995; Blazquez et al., 2007; Büttner-Ennever and Horn, 2014).

### Functional Significance of CR in Premotor Upgaze Pathways

As already discussed previously, CR is present only in a subgroup of premotor neurons involved in upgaze. For example, the excitatory secondary vestibulo-ocular projections from the magnocellular parts of the medial and superior vestibular nuclei linking the anterior semicircular canals to the extraocular muscles (mediating compensatory upward movements after stimulation of the anterior canal) do not contribute to the CR-projection to upgaze motoneurons (Ahlfeld et al., 2011). This also applies to humans, where CR-positive neurons have mainly been found in an area of the medial vestibular nucleus termed the “calretinin area” (Baizer and Broussard, 2010; Baizer et al., 2013), which is not the location of secondary vestibular neurons (Büttner-Ennever and Gerrits, 2004; Büttner-Ennever and Horn, 2014).

The functional significance of the CR expression in premotor neurons mediating upgaze is not clear. Whereas the presence of PAV is usually connected to highly active and fast-firing neurons such as those of the saccadic system, which are often ensheathed by perineuronal nets (Härtig et al., 1995; Horn et al., 2003a), the expression of CR in certain cell groups is less clear. In general calcium-binding proteins serve as Ca^2+^ buffers, which control the duration and spread of Ca^2+^ signals, and Ca^2+^ sensors, which translate Ca^2+^ concentration changes into specific intracellular signals (Schwaller, 2009; Brini et al., 2014). Proposed functions of CR involve a role in neuroprotection, development and regulation of neuronal excitability (for review, see Schwaller, 2014). Although up- and down-burst neurons in the RIMLF exhibit similar firing characteristics, which may be reflected by the common expression of PAV, up-burst neurons contain an additional calcium-binding protein indicating a different Ca^2+^ control mechanism (Horn et al., 2003a; Zeeh et al., 2013). Up- and downward saccades can be impaired in several clinical conditions. Structural lesions caused by infarcts or tumors may affect the efferent pathways of burst neurons, which differ for up- and downgaze as discussed previously (Zeeh et al., 2013). There are neurodegenerative diseases like progressive supranuclear palsy (PSP) or Niemann-Pick disease type C (NPC) that are characterized by paresis of vertical saccades often affecting only one direction initially (Chen et al., 2010; Strupp et al., 2014). Since the disturbance of the Ca^2+^ signaling mechanism compromises normal neuronal function, it may represent one mechanism for the cause of neurodegeneration as suggested for Parkinson’s disease or ataxias caused by an effect on the Purkinje cells (Brini et al., 2014). This is supported by the clinical observation that in NPC downward saccades are impaired first. With CR as a marker protein for putative excitatory premotor saccadic burst neurons in RIMLF and INC and smooth pursuit neurons in the y-group these populations can be specifically identified for analysis in future post-mortem analysis of cases with NPC.

## Conclusion

Given the similarities in the organization and histochemical properties of functional cell groups of the oculomotor system in the monkey and humans (Horn et al., 1995; Horn and Büttner-Ennever, 1998; Horn and Adamczyk, 2011) it is likely that the CR-positive neurons identified in RIMLF, INC and the y-group represent excitatory premotor neurons driving upgaze. The histological identification of premotor neurons involved in up- or downgaze allows the analysis of these cell groups on a cellular level. Furthermore, immunostaining for CR, in combination with PAV or ACAN provides a histological tool for a specific analysis of premotor up and down neurons in future post-mortem studies on cases with PSP and NPC.

## Author Contributions

Acquisition of the data and analysis with preparation of the figures was performed by CA. Conception of the work was done by CA and AH. Interpretation of the data, writing and revising the manuscript and final approval of the manuscript was done by CA, MS, KJ, AH.

## Funding

The study was supported by FoFöLe 697, Bundesministerium für Bildung und Forschung (IFBLMU 01EO0901, Brain-Net-01GI0505), Deutsche Forschungsgemeinschaft (HO1639/4-4).

## Conflict of Interest Statement

The authors declare that the research was conducted in the absence of any commercial or financial relationships that could be construed as a potential conflict of interest.

## Abbreviations

3: third ventricle
ACAN: aggrecan
ARC: arcuate nucleus
nIII: oculomotor nucleus
nIV: trochlear nucleus
nVI: abducens nucleus
CCN: central caudal nucleus
CEN: central group
ChAT: acetylcholine transferase
CR: calretinin
DCN: dorsal cochlear nucleus
DEN: dentate nucleus
DVN: descending vestibular nucleus
EAP: extravidin-peroxidase
EWcp: Edinger-Westphal nucleus, central projecting part
GABA: gamma-aminobutyric acid
GAD: glutamate decarboxylase
ICP: inferior cerebellar peduncle
INC: interstitial nucleus of Cajal
IO: IO muscle
IR: inferior rectus muscle
LP: levator palpebrae muscle
MB: mammillary body
MLF: medial longitudinal fascicle
MR: medial rectus muscle
MT: mammillothalamic tract
MVN: medial vestibular nucleus
NIII: oculomotor nerve
nIII: oculomotor nucleus
nIV: trochlear nucleus
nVI: abducens nucleus
PAG: periaqueductal gray
PAV: parvalbumin
PC: posterior commissure
PO: principal inferior olive
PPH: prepositus hypoglossal nucleus
PT: pyramidal tract
RIMLF: rostral interstitial nucleus of the medial longitudinal fascicle
RN: red nucleus
SC: superior colliculus
SOA: supraoculomotor area
SOL: solitary nucleus
SpV: spinal trigeminal nucleus
SR: superior rectus muscle
ST: subthalamicus
TR: tractus retroflexus
TSpV: spinal trigeminal tract
VCN: ventral cochlear nucleus.
